# The Impact of Concierge Pediatric Care on Parental Anxiety and Health-Seeking Behavior in Infancy

**DOI:** 10.7759/cureus.117392

**Published:** 2026-10-03

**Authors:** Sahar Miri, Matthew Araujo, Anuradha Ramdas, Natalie Magagna, Chad Rudnick

**Affiliations:** 1 Dr. Kiran C. Patel College of Osteopathic Medicine, Nova Southeastern University, Fort Lauderdale, USA; 2 Department of Biomedical Sciences, Charles E. Schmidt College of Medicine, Florida Atlantic University, Boca Raton, USA; 3 Department of Public Health, Miller School of Medicine, University of Miami, Miami, USA; 4 Charles E. Schmidt College of Science, Florida Atlantic University, Boca Raton, USA; 5 Dr. Pallavi Patel College of Health Care Sciences, Nova Southeastern University, Boca Raton, USA; 6 Charles E. Schmidt College of Medicine, Florida Atlantic University, Boca Raton, USA

**Keywords:** concierge pediatric care, cross-sectional survey, direct primary care, healthcare-seeking behavior, online health information, parental anxiety, pediatric well-child visits, provider access

## Abstract

Background and objective

The early months of a child’s life are often marked by heightened parental anxiety, particularly regarding health concerns. With growing access to online health information and the rise of concierge pediatric practices, it is important to understand how these factors influence parental anxiety and healthcare-seeking behavior. This study aimed to evaluate the relationship between internet use and parental anxiety, assess the impact of concierge pediatric care on stress, and examine how ease of access affects both online and in-person visit frequency during a child’s first year.

Methods

A cross-sectional survey was administered to parents of children aged nine to 18 months enrolled in a concierge pediatric practice. The survey assessed demographics, primary concerns, visit frequency, online information-seeking behaviors, and the perceived impact of concierge care on stress and healthcare use. Statistical analyses included chi-square goodness-of-fit and independence tests, Mann-Whitney U, and Kruskal-Wallis H tests.

Results

Most parents reported reduced stress due to the availability of concierge care for their child, with 76.7% indicating greater-than-expected reductions and 81.4% reporting an overall decrease in anxiety. Satisfaction with pediatrician availability was significantly associated with reduced stress (χ² = 4.75, p = 0.029). Ease of access influenced behavior (χ² = 6.14, p = 0.046), with nearly half reporting fewer visits. No significant associations were found between anxiety and parent education, membership duration, internet use, or number of children.

Conclusions

Concierge pediatric care was associated with reduced parental anxiety and more efficient healthcare utilization. Provider access appears to be central to these outcomes.

## Introduction

Becoming a parent brings a major emotional and psychological shift, often filled with stress and worry, especially when it comes to an infant’s health [1]. Systematic reviews have shown that parental anxiety is associated with adverse emotional and behavioral outcomes in children, with effects emerging in infancy and intensifying throughout development [2,3]. In seeking reassurance, many new parents turn to doctors, nurses, and increasingly the internet to help ease their concerns. Social media platforms and online communities have become common sources of support, often consulted before and after clinical encounters [4]. Qualitative work also demonstrates that parents of infants and toddlers commonly turn to online health resources because of their accessibility and immediacy [5].

However, while digital health content is more accessible than ever, it can sometimes create confusion or even heighten anxiety when advice is inconsistent or inaccurate [4]. Recent research indicates that parental anxiety is often accompanied by excessive online searching for health-related information, a behavior strongly associated with heightened stress and psychological distress [6]. These effects appear particularly pronounced among parents experiencing elevated levels of loneliness or pre-existing anxiety [7]. Furthermore, not all sources are trustworthy, and distinguishing reliable content from misinformation can be challenging without guidance or training [8]. This may often lead to the unintentional acceptance of online misinformation or disinformation as fact, which can be extremely harmful to patients and families [9].

In response to these challenges, concierge pediatric care or direct primary care has gained traction as an alternative approach. For the purposes of this paper, the term concierge pediatric care will be used to refer to both concierge and direct primary care models. These practices offer families greater access to their pediatricians, including after-hours availability, more personalized care, and quicker communication, which may help reduce the uncertainty that often accompanies early parenthood.

Recent literature reviews have highlighted the potential benefits of concierge medicine, including enhanced patient satisfaction, improved access, and increased preventive service utilization [10,11]. Still, there has been a paucity of research into how this model impacts parents, specifically in terms of their anxiety levels, use of online health resources, and how frequently they seek medical care for their child.

This study examines how concierge or direct primary care pediatric services may shape parents' experiences during their child’s first year of life. It focuses on how this type of care impacts parental anxiety, trust in internet-based information, and how frequently they use in-person or virtual visits. By exploring these factors, the research aims to understand the relationships between concierge healthcare and parental anxiety, while supporting more thoughtful, parent-focused improvements in pediatric care.

This article was previously presented as a Power Talk presentation at the Medical Student Research Forum of the Future of Pediatric Practice Conference in Orlando, Florida, on August 30, 2025.

## Materials and methods

Study design and participants

This study presents a retrospective account of an internal quality-improvement (QI) survey initiative conducted independently at a single concierge pediatric practice, examining self-reported parental anxiety, health concerns, and information-seeking behavior during a child’s first 18 months of life, with a focus on concierge pediatric care access. The research team was directly involved in the practice's QI initiative, including designing the survey instrument used to collect this internal quality assurance (QA) data. Participants were parents or legal guardians of infants aged nine to 18 months receiving care at the practice. All eligible parents attending well-child visits between October 2024 and March 2025 were invited to complete an anonymous, voluntary survey during a brief interval after vital signs were taken and while the pediatrician prepared growth charts and materials outside the room.

There were a total of 43 participants. Only fully completed surveys were included in individual analyses, resulting in slight variation in sample size across different statistical tests. A small number of participants began the survey but did not complete it due to common disruptions during well-child visits and were therefore excluded from analysis.

Data collection

A 19-item paper-based questionnaire was developed by the research team and distributed in the clinic while patients waited for their physician (Appendices). Questionnaires were collected before the end of each visit. The survey included multiple-choice and Likert-scale items addressing baseline parental anxiety (self-rated), perceived impact of concierge care on stress/anxiety, satisfaction with pediatrician availability, behavioral changes in healthcare utilization (e.g., frequency of visits), and demographics and practice usage characteristics (e.g., number of children and membership duration).

Measures

Parental anxiety was assessed using a study-specific five-point Likert scale (1 = not anxious at all to 5 = extremely anxious), modeled on previous research employing brief self-report measures of parental anxiety [12-14]. The anxiety-related questions echo the conceptual basis of validated instruments such as the Parental Attitudes, Beliefs, and Understanding of Anxiety (PABUA) that capture parental emotional response via Likert‑style ratings [15]. Internet usage frequency was categorized as never, rarely, sometimes, often, or always. Pediatrician availability was determined by reported access to 24/7 services, and visit frequency was counted for both in-person and telehealth encounters.

Data handling and analysis

Survey responses were exported to Microsoft Excel (Microsoft Corporation, Redmond, WA) and analyzed using R statistical software, version 4.5.0 (R Foundation for Statistical Computing, Vienna, Austria). Descriptive statistics, including frequencies, percentages, means, and standard deviations, were used to summarize participant characteristics and response distributions. Chi-square goodness-of-fit tests were applied to assess whether observed response distributions differed significantly from a uniform distribution for questions evaluating perceived outcomes, behavioral changes, and demographic patterns (e.g., Q17, Q18, and Q19). Chi-square tests of independence were used to examine associations between categorical variables, such as satisfaction with pediatrician availability and reported anxiety reduction. Nonparametric tests, including the Mann-Whitney U test and Kruskal-Wallis H test, were used to compare non-normally distributed variables across groups (e.g., encounter frequency by first-time vs. experienced parents or by stress level). Only complete, pairable responses were included in bivariate analyses. A significance threshold of P < 0.05 was used for all statistical tests.

Ethical considerations

Participation was voluntary and anonymous. The study involved no personally identifiable information and posed minimal risk to participants. Nova Southeastern University’s (NSU) Institutional Review Board (IRB) reviewed the activity. It determined that it was conducted outside NSU’s oversight and did not constitute research subject to NSU IRB review, as it met criteria for internal quality improvement and program evaluation rather than human-subjects research intended to generate generalizable knowledge. Correspondence documenting this determination is available upon request. Ethical principles of informed consent, confidentiality, and data integrity were followed throughout data collection and analysis.

## Results

Participant demographics

A total of 43 parents participated in the study. Full demographic characteristics are presented in Table 1. The majority (55.8%, n = 24) were between 32 and 38 years of age, followed by 23.3% (n = 10) between 25 and 31 years, 16.3% (n = 7) between 39 and 45 years, and 4.7% (n = 2) aged 46 years or older. In terms of educational attainment, 48.8% (n = 21) held a bachelor’s or associate degree, 41.9% (n = 18) had a master’s, doctoral, or professional degree, 7.0% (n = 3) had some college experience, and 2.3% (n = 1) had some post-baccalaureate education without a degree. Nearly one-third of participants (30.2%, n = 13) were first-time parents. Among the 30 parents who were not first-time parents, the average number of other children was 1.27 (SD = 0.52); most reported one additional child (76.7%, n = 23), followed by two (20.0%, n = 6) and three or more (3.3%, n = 1). Regarding concierge membership, 34.9% (n = 15) had been enrolled for two to three years, 30.2% (n = 13) for 12 to 18 months, 18.6% (n = 8) for over four years, 11.6% (n = 5) for six to 12 months, and 4.7% (n = 2) for less than six months.

**Table 1:** Participant demographics (N = 43, unless otherwise noted).

Variable	n	%
Child age		
9-11 months	13	30.2%
12-14 months	5	11.6%
15-18 months	25	58.1%
Parent/guardian age		
25-31 years	10	23.3%
32-38 years	24	55.8%
39-45 years	7	16.3%
46+ years	2	4.7%
Relationship to child		
Mother	32	74.4%
Father	2	4.7%
Both parents	7	16.3%
Not reported	2	4.7%
Highest level of education		
High school	0	0.0%
Some college	3	7.0%
Bachelor's or associate degree	21	48.8%
Some post-baccalaureate	1	2.3%
Master's, doctoral, or professional degree	18	41.9%
First-time parent		
Yes	13	30.2%
No	30	69.8%
Number of other children (among non-first-time parents, n = 30)		
One	23	76.7%
Two	6	20.0%
Three or more	1	3.3%
Concierge membership duration		
Less than 6 months	2	4.7%
6-12 months	5	11.6%
12-18 months	13	30.2%
2-3 years	15	34.9%
4+ years	8	18.6%
Child born into the concierge practice		
Yes	37	86.0%
No	6	14.0%
Previously at a different practice		
Yes	12	27.9%
No	31	72.1%

Primary areas of concern

Parents reported a range of concerns during their child’s first year (Table 2); this item allowed multiple selections, so percentages do not sum to 100%. Among the 42 parents who answered this item, the most commonly cited concern was general health (e.g., fever and cold) (64.3%, n = 27), followed by developmental milestones (35.7%, n = 15), vaccinations and side effects (21.4%, n = 9), sleep issues (21.4%, n = 9), feeding or nutrition concerns (19.0%, n = 8), and other concerns (16.7%, n = 7). These results reflect the diverse sources of parental anxiety, encompassing both developmental expectations and medical uncertainty.

**Table 2:** Primary areas of parental concern during the child’s first year, among the 42 parents who answered this item (select all that apply; percentages do not sum to 100%).

Concern	n	%
General health concerns	27	64.3%
Developmental milestones	15	35.7%
Vaccinations and side effects	9	21.4%
Sleep issues	9	21.4%
Feeding/nutrition concerns	8	19.0%
Other (including free-text response “Everything!”)	7	16.7%

Healthcare utilization

The average number of pediatric encounters, both in person and virtual, in the past year was 21.1 (SD = 9.8, n = 41), and the average total number of encounters across membership duration was 32.7 (n = 41). Notably, of the 42 parents who answered this item, half reported that the ease of access provided by the concierge model led them to visit the pediatrician in-office less frequently (50.0%, n = 21), while 31.0% (n = 13) reported visiting more often, and 19.0% (n = 8) saw no change in behavior (Table 3). This distribution was statistically significant, χ²(2, N = 42) = 6.14, p = 0.046, suggesting that improved access may reduce unnecessary in-person visits. Additionally, first-time parents (n = 13) had a higher median number of pediatric encounters in the past year than experienced parents (n = 28) (26 vs. 17), and the difference was statistically significant (Mann-Whitney U = 258.5, p = 0.033).

**Table 3:** Healthcare utilization and impact of concierge pediatric care on parental anxiety, with associated test statistics.

Variable/Comparison	Category	n (%) or median	Test statistic (p-value)
Ease of access effect on in-office visit frequency (Q19, N = 42)	Visited less frequently	21 (50.0%)	χ²(2) = 6.14, p = 0.046
	Visited more frequently	13 (31.0%)	
	No change	8 (19.0%)	
Pediatrician's effect on anxiety vs. expectations (Q17, N = 43)	Significantly reduced	33 (76.7%)	χ²(2) = 38.70, p < 0.001
	Somewhat reduced	9 (20.9%)	
	No impact	1 (2.3%)	
Overall impact of concierge care on anxiety (Q18, N = 43)	Significantly lowered	35 (81.4%)	χ²(2) = 45.95, p < 0.001
	Slightly lowered	7 (16.3%)	
	No change	1 (2.3%)	
Satisfaction with pediatrician availability (Q16, N = 43)	Extremely satisfied	41 (95.3%)	χ²(1) = 35.37, p < 0.001
	Moderately satisfied	2 (4.7%)	
Satisfaction (Q16) × anxiety reduction (Q18), N = 43	Extremely satisfied – reduced anxiety	41	χ²(1) = 4.75, p = 0.029
	Extremely satisfied – no reduction	0	
	Moderately satisfied – reduced anxiety	1	
	Moderately satisfied – no reduction	1	
Child born into the concierge practice (Q8, N = 43)	Yes	37 (86.0%)	χ²(1) = 22.35, p < 0.001
	No	6 (14.0%)	
Number of additional children, non-first-time parents (Q6, N = 30)	One	23 (76.7%)	χ²(2) = 26.60, p < 0.001
	Two	6 (20.0%)	
	Three or more	1 (3.3%)	
Internet consultation frequency (Q14, N = 43)	Never	2 (4.7%)	χ²(4) = 17.12, p = 0.002
	Rarely	10 (23.3%)	
	Sometimes	18 (41.9%)	
	Often	8 (18.6%)	
	Always	5 (11.6%)	
Effect of online resources on anxiety among users (Q15, N = 42)	Increased anxiety	20 (47.6%)	χ²(3) = 13.81, p = 0.003
	No effect	11 (26.2%)	
	Decreased anxiety	7 (16.7%)	
	Did not use online resources	4 (9.5%)	
Pediatric encounters in past year, by parent status	First-time parents (n = 13)	26 (median)	Mann-Whitney U = 258.5, p = 0.033
	Experienced parents (n = 28)	17 (median)	
Pediatric encounters in past year, by overall anxiety-impact category (Q18, N = 41)	Significantly lowered (n = 33)	21 (median)	Kruskal-Wallis H(2) = 0.57, p = 0.76
	Slightly lowered (n = 7)	26 (median)	
	No change (n = 1)	25 (median)	

Impact of concierge pediatric care on parental anxiety

To evaluate the effect of concierge pediatric care on anxiety, two related questions were analyzed using chi-square goodness-of-fit tests (Table 3). For question 17, which assessed how the pediatrician affected anxiety/stress compared to expectations, the majority reported a “Significantly reduced” effect (76.7%, n = 33), followed by “Somewhat reduced” (20.9%, n = 9) and “No impact” (2.3%, n = 1). The distribution was highly significant, χ²(2, N = 43) = 38.70, p < 0.001. Similarly, for question 18 (overall impact on anxiety), responses were skewed toward improvement, with 81.4% (n = 35) reporting a significantly lowered stress level, 16.3% (n = 7) reporting a slight reduction, and 2.3% (n = 1) reporting no change, χ²(2, N = 43) = 45.95, p < 0.001.

Satisfaction with pediatrician availability (Q16) was significantly associated with anxiety outcomes (Q18). Nearly all parents rated their satisfaction as “Extremely satisfied” (95.3%, n = 41), with the remainder “Moderately satisfied” (4.7%, n = 2); this distribution was itself highly skewed, χ²(1, N = 43) = 35.37, p < 0.001. All 41 parents who were “Extremely satisfied” also reported a reduction in anxiety, compared to only one of the two parents who were “Moderately satisfied.” This association was significant, χ²(1, N = 43) = 4.75, p = 0.029 (Table 3).

Contextual and non-significant findings

Several additional contextual patterns emerged (Table 3). Most parents reported that their child was born into the concierge practice where the study was completed (86.0%, n = 37 vs. 14.0%, n = 6), χ²(1, N = 43) = 22.35, p < 0.001. Among the 30 parents who were not first-time parents, the number of additional children was distributed non-uniformly (one child: 76.7%, n = 23; two: 20.0%, n = 6; three or more: 3.3%, n = 1), χ²(2, N = 30) = 26.60, p < 0.001. Responses to questions about digital behavior were also significantly non-uniform: the frequency of internet consultations (Q14) ranged from “Sometimes” (41.9%, n = 18) to “Never” (4.7%, n = 2), χ²(4, N = 43) = 17.12, p = 0.002, and the perceived effect of online resources on stress (Q15) was most often “Increased” (47.6%, n = 20) among the 42 parents who used online resources, χ²(3, N = 42) = 13.81, p = 0.003, indicating variability in how families used and responded to online health information.

However, these digital variables were not significantly associated with anxiety reduction outcomes. Similarly, several demographic and care-related variables were not significantly related to reported stress outcomes. These included parent education level, membership duration, first-time parent status, whether the child was born into the concierge practice, and the number of encounters in the past year (all p > 0.05). Notably, encounter frequency was not associated with reported stress levels (Kruskal-Wallis H(2) = 0.57, p = 0.76).

## Discussion

This study aimed to evaluate the perceived impact of concierge pediatric or direct primary care on parental anxiety and associated healthcare behaviors. The findings demonstrate a clear and statistically significant pattern: the vast majority of parents reported that having a concierge pediatrician reduced their anxiety, with many indicating that the effect exceeded expectations. These results support prior research linking parental emotional health to perceived provider responsiveness and system-level support [16,17]. Our findings contribute to existing literature showing that parenting stress may not only affect individual well-being but can also have broader consequences for healthcare access and utilization [18].

Both the comparative question (Q17) and the overall outcome question (Q18) yielded distributions highly skewed toward anxiety reduction, with p-values well below conventional thresholds. One of the most compelling explanatory factors was satisfaction with pediatrician availability (Q16), which was significantly associated with reported anxiety reduction. In this practice, a member of the pediatric care team (physician or nurse practitioner) is available to the patient/parent 24 hours per day. There is no call-triage service, but rather all encounters are directed most often to the child’s primary pediatrician, or in the case of a pediatrician’s day off, a covering provider within the practice is assigned to cover the panel of patients. Nearly all parents who reported being “Extremely satisfied” also indicated a decrease in anxiety. This supports the hypothesis that accessibility and responsiveness of the pediatric practice, which are hallmarks of concierge care, rather than outside triage, “on-call services”, or third-party telemedicine platforms, enhance parental confidence and peace of mind during the early stages of parenting [16,19].

Beyond psychological outcomes, this study also captured changes in healthcare-seeking behavior. When asked whether ease of access affected how often they sought care (Q19), parents most commonly reported fewer in-person visits. This suggests that improved telehealth accessibility may reduce unnecessary inpatient appointments, possibly due to increased reassurance or efficient resolution of concerns via direct communication (e.g., secure messaging and phone calls). Our findings align with previous research showing that parents particularly value convenience, credibility, and ease of access when seeking health information and support for their children [5].

Survey responses also contextualized family dynamics. Most parents indicated their child was born into the concierge practice where the study was performed, and that they had more than one child. These factors may support long-term trust and continuity of care. While internet usage varied among respondents, it was not significantly related to anxiety outcomes. This contrasts with literature noting that online misinformation and fragmented digital advice can increase parental distress [4,17]. In this study, real-time access to a trusted provider may have mitigated that effect.

Limitations

While these results are promising, several limitations warrant consideration. The study was conducted at a single concierge pediatric practice located in a suburban area, which may reflect practice-specific culture or systems that are not generalizable to other models. The sample size was modest, and strongly positive response patterns limited statistical power for subgroup comparisons. The survey employed a cross-sectional design, which precludes causal inferences.

Additionally, the education level of participants was notably higher than national averages. Over 90% of parents in the sample held a college or graduate degree, compared to just 58% of households with children under 18 in the US with at least one adult holding a bachelor’s degree [20]. This may bias the results toward higher health literacy, trust in providers, or resource access. Even with higher educational attainment, participants may still be susceptible to online pediatric health misinformation [21]. Future research should recruit more demographically diverse participants to determine whether similar outcomes occur across broader populations.

## Conclusions

Concierge pediatric care appears to be associated with reduced reported parental anxiety and more efficient use of pediatric services. High satisfaction with provider access was a central factor in both psychological reassurance and visit behavior. These results highlight the potential of concierge care to enhance emotional and logistical support for parents, particularly in early parenthood. Further research with larger and more diverse samples is needed to explore the broader applicability and long-term outcomes of this model of care.

## Appendices

Parental anxiety, healthcare behavior, and information-seeking questionnaire

This survey was administered to parents of infants aged nine to 18 months enrolled in a concierge pediatric practice. It was developed to assess parental anxiety, healthcare utilization patterns, and information-seeking behavior during a child’s first year of life.

Part 1: General Information

**Table 4:** Questionnaire Part 1: General information.

#	Question	Response options
1	Child age:	9–11 months; 12–14 months; 15–18 months
2	Parent/guardian age:	18–24; 25–31; 32–38; 39–45; 46+
3	Relationship to child:	mother; father; guardian
4	Highest level of education:	high school; some college; bachelor or associate degree; some post-baccalaureate; master’s or doctorate degree
5	Is this your first child?	yes; no
6	If not, how many other children do you have?	one; two; three or more
7	How long have you been a member of VIPediatrics?	less than 6 months; 6–12 months; 12–18 months; 2–3 years; 4+ years
8	Was your child born into VIPediatrics?	yes; no
9	Were you at a different practice before joining VIPediatrics?	yes; no

Part 2: Anxiety/Stress Levels During Your Child’s First Year

**Table 5:** Questionnaire Part 2: Anxiety/stress levels during your child’s first year.

#	Question	Response options
10	How often did you feel anxious or stressed about your child’s health, on a scale of 1 (not anxious at all) to 5 (extremely anxious)?	1; 2; 3; 4; 5
11	Primary cause(s) of concern (select all that apply):	general health concerns; sleep issues; feeding/nutrition; developmental milestones; vaccinations/side effects; other (specify)
12	Confidence in managing your child’s health:	very confident; somewhat confident; neutral; somewhat unsure; very unsure

Part 3: Use of Pediatrician and Online Resources

**Table 6:** Questionnaire Part 3: Use of pediatrician and online resources.

#	Question	Response options
13	How often did you consult your pediatrician outside of well visits?	weekly; monthly; a few times during the year; rarely; never
14	How often did you consult online resources before contacting your pediatrician?	always; often; sometimes; rarely; never
15	Effect of online resources on anxiety:	increased my anxiety; decreased my anxiety; no effect; I did not use online resources
16	Satisfaction with pediatrician availability:	extremely satisfied; moderately satisfied; neutral; moderately dissatisfied; extremely dissatisfied
17	Impact of concierge care on anxiety compared to expectations:	significantly reduced; somewhat reduced; no impact; increased

Part 4: Overall Impact on Anxiety and Behavior

**Table 7:** Questionnaire Part 4: Overall impact on anxiety and behavior.

#	Question	Response options
18	Overall impact of concierge pediatric care:	significantly lowered; slightly lowered; no change; increased; not applicable
19	Was your healthcare behavior influenced by ease of access?	yes, I visited less frequently; yes, I visited more frequently; no change; I’m not sure

20. Additional comments: (open-ended response)

## References

[1] (2026). National Alliance on Mental Illness (NAMI). Guide to maternal & new parent mental health. https://www.nami.org/your-journey/maternal-new-parent-mental-health/.

[2] Rees S, Channon S, Waters CS (2019). The impact of maternal prenatal and postnatal anxiety on children's emotional problems: a systematic review. Eur Child Adolesc Psychiatry.

[3] Sweeney S, Wilson C (2023). Parental anxiety and offspring development: a systematic review. J Affect Disord.

[4] Frey E, Bonfiglioli C, Brunner M, Frawley J (2022). Parents' use of social media as a health information source for their children: a scoping review. Acad Pediatr.

[5] Hendry D, Straker L, Bourne B, Coshan S, Kumwembe N, McCarthy C, Zabatiero J (2024). Parental practices and perspectives on health and digital technology use information seeking for children aged 0-36 months. Health Promot J Austr.

[6] Barke A, Doering BK (2020). Development of an instrument to assess parents’ excessive web-based searches for information pertaining to their children’s health: the “Children’s Health Internet Research, Parental Inventory” (CHIRPI). J Med Internet Res.

[7] Onishi R (2024). Parenting-related social networking site use and psychological distress in parents of infants: cross-sectional study exploring the moderating effects of loneliness and parenting anxiety. JMIR Pediatr Parent.

[8] Ameenuddin N, Thompson LA (2025). What parents need to know about online misinformation. JAMA Pediatr.

[9] Ashby J (2026). Ashby J. The effects of medical misinformation on the American public. https://scholarsarchive.byu.edu/cgi/viewcontent.cgi?params=/context/ballardbrief/article/1123/&path_info=07_The_Effects_of_Medical_Misinformation_on_the_American_Public___Ballard_Brief.pdf.

[10] Rylands KS, Collins CM, Collins DR Jr (2025). Maximizing the value of concierge medicine: a systematic review of cost, access, and outcomes. Am J Med.

[11] Alhawshani S, Khan S (2024). A literature review on the impact of concierge medicine services on individual healthcare. J Family Med Prim Care.

[12] Di Renzo M, Bianchi di Castelbianco F, Vanadia E, Petrillo M, Racinaro L, Rea M (2020). Parental perception of stress and emotional-behavioural difficulties of children with autism spectrum disorder and specific language impairment. Autism Dev Lang Impair.

[13] Shek DT, Li X, Yang B, Yang J (2025). Is parental anxiety related to child anxiety? Insights from a four-wave longitudinal study in a Chinese context. Front Psychiatry.

[14] Burstein M, Ginsburg GS, Tein JY (2010). Parental anxiety and child symptomatology: an examination of additive and interactive effects of parent psychopathology. J Abnorm Child Psychol.

[15] Wolk CB, Caporino NE, McQuarrie S (2016). Parental Attitudes, Beliefs, and Understanding of Anxiety (PABUA): development and psychometric properties of a measure. J Anxiety Disord.

[16] Taubman-Ben-Ari O, Ben-Yaakov O, Chasson M (2021). Parenting stress among new parents before and during the COVID-19 pandemic. Child Abuse Negl.

[17] Kubb C, Foran HM (2020). Online health information seeking by parents for their children: systematic review and agenda for further research. J Med Internet Res.

[18] Campisi C, Pham D, Rapoport E, Adesman A (2024). Parenting stress, community support, and unmet health care needs of children in the US. Matern Child Health J.

[19] Lee TJ, Guzy J, Johnson D, Woo H, Baraff LJ (2002). Caller satisfaction with after-hours telephone advice: nurse advice service versus on-call pediatricians. Pediatrics.

[20] (2025). National Center for Education Statistics. Characteristics of children’s families. https://nces.ed.gov/programs/coe/indicator/cce/family-characteristics.

[21] Scherer LD, McPhetres J, Pennycook G (2021). Who is susceptible to online health misinformation? A test of four psychosocial hypotheses. Health Psychol.

